# The effect of core and lanthanide ion dopants in sodium fluoride-based nanocrystals on phagocytic activity of human blood leukocytes

**DOI:** 10.1007/s11051-017-3779-9

**Published:** 2017-02-13

**Authors:** Bartlomiej Sojka, Aurelia Liskova, Miroslava Kuricova, Mateusz Banski, Jan Misiewicz, Maria Dusinska, Mira Horvathova, Silvia Ilavska, Michaela Szabova, Eva Rollerova, Artur Podhorodecki, Jana Tulinska

**Affiliations:** 1 Department of Experimental Physics, Wroclaw University of Science and Technology, Wyb. Wyspianskiego 27, 50-370 Wroclaw, Poland; 2 Medical Faculty, Department of Immunology and Immunotoxicology, Slovak Medical University, Limbova 12, 833 03 Bratislava, Slovakia; 3 Health Effects Laboratory, Department of Environmental Chemistry, Norwegian Institute for Air Research, Instituttveien 18, 2027 Kjeller, Norway; 4 Faculty of Public Health, Department of Toxicology, Slovak Medical University, Limbova 12, 833 03 Bratislava, Slovakia

**Keywords:** Nanocrystals, β-NaLnF4, Hydrophilic, Toxicity, Phagocytes, Lanthanide ions, Nanomedicine, Health effects

## Abstract

Sodium fluoride-based β-NaLnF4 nanoparticles (NPs) doped with lanthanide ions are promising materials for application as luminescent markers in bio-imaging. In this work, the effect of NPs doped with yttrium (Y), gadolinium (Gd), europium (Eu), thulium (Tm), ytterbium (Yb) and terbium (Tb) ions on phagocytic activity of monocytes and granulocytes and the respiratory burst was examined. The surface functionalization of <10-nm NPs was performed according to our variation of patent pending ligand exchange method that resulted in meso-2,3-dimercaptosuccinic acid (DMSA) molecules on their surface. Y-core-based NCs were doped with Eu ions, which enabled them to be excited with UV light wavelengths. Cultures of human peripheral blood (*n* = 8) were in vitro treated with five different concentrations of eight NPs for 24 h. In summary, neither type of nanoparticles is found toxic with respect to conducted test; however, some cause toxic effects (they have statistically significant deviations compared to reference) in some selected doses tested. Both core types of NPs (Y-core and Gd-core) impaired the phagocytic activity of monocytes the strongest, having minimal or none whatsoever influence on granulocytes and respiratory burst of phagocytic cells. The lowest toxicity was observed in Gd-core, Yb, Tm dopants and near-infrared nanoparticles. Clear dose-dependent effect of NPs on phagocytic activity of leukocytes and respiratory burst of cells was observed for limited number of samples.

## Introduction

In recent years, many research groups (including ours) have used sodium fluoride-based nanoparticles (NPs) doped with lanthanide ions for application as luminescent markers in bio-imaging (Chen et al. 2012; Hou et al. 2013; Liu et al. 2013; Sudheendra et al. 2014; Cui et al. 2015; Ma et al. 2015). While they possess the desired optical properties for this purpose (i.e. excellent photostability, hardly any luminescence bleaching and blinking, facile emission tuning), there is still one issue that needs to be addressed when it comes to administering them to living organisms, namely their toxicity. There are some reports evaluating the potential toxic effects of sodium-based NPs. They are focused on one type of NPs (e.g. up-converting) or they include only one typical cytotoxicity test (e.g. MTT assay) (Yang et al. 2013; Zeng et al. 2015; Hu et al. 2016). Among these examples (and a few reports cited later), there are none that deal with the immunological response upon introduction of NPs.

In this work, we examine the effect of sodium fluoride-based NPs doped with yttrium (Y), gadolinium (Gd), europium (Eu), thulium (Tm), ytterbium (Yb) and terbium (Tb) ions on phagocytosis, which is considered among first-line responders after exposure to NPs. Unlike quantum dots, which are often composed of cadmium, sodium fluoride-based nanocrystals (NCs) are made of non-toxic elements. Therefore, they are an excellent host matrix from bioapplication point of view. Sodium fluoride matrix also has beneficial effects on optical properties of lanthanides doped in them. That is why NPs with rare earth ions are a promising candidate for probes in bioimaging. What is more, NCs can be designed to be an up-converting marker. It means that we excite them in the near infrared (NIR) spectral region, and they will emit in visible (VIS) range, in this case giving green colour. NIR excitation is important because the light of these wavelengths is the least absorbed by the biological tissue, which means most of it reaches the NCs and is not diminished earlier. Jacques (2013) examined different types of structures that are present in living organism. From this research, one can see that indeed the absorption spectra have a distinct minimum in the 800–1100-nm rage. That is why, NIR excitation is one of the biggest advantages of β-NaLnF_4_ doped NCs over other biomarkers like fluorescent proteins and organic dyes.

In the previous studies, we have demonstrated that hydrophobic sodium fluoride-based NCs do not induce toxic effects in low concentrations and in some cases even in high doses. Moreover, it is stated that the main reason for toxicity in those samples was connected with their solvent, namely cyclohexane (Sojka et al. 2014). Therefore, a pressing matter occurred to make the NCs hydrophilic and to be able to solve them in water.

In this study, experiments are focused on assessing the effect of NCs on the function of phagocytic cells in human peripheral blood. Phagocytosis is an important mechanism of non-specific (natural) immunity to defend the organism against pathogens and to remove cell debris. Professional phagocytes are immune cells that can ingest 0.5–10-μm-sized particles such as bacteria, virus, fungi or dust. Mononuclear phagocytes are derived from myeloid progenitor cells in bone marrow and over time developed into granulocytes and monocytes. Granulocytes are differentiated into neutrophil, eosinophil and basophil granulocytes. The phagocytosis of invading bacteria is the main role of neutrophil granulocytes (Fröhlich 2015). Monocytes circulate in the blood and differentiate into macrophages in the tissue. In response to NP exposure, macrophages in particular are believed to be among the “first responders” and primary cell types that uptake and process NPs, mediating host biological responses by subsequent interactions with inflammatory signalling pathways and immune cells (Herd et al. 2015).

The phagocytic activity can be evaluated using fluorescein-labelled bacteria (for example *Staphylococcus aureus*) in monocytes and granulocytes exposed to the NPs. Respiratory burst (reactive oxygen production) can be performed in granulocytes by the detection of reactive oxygen species (ROS), which is produced upon phagocytosis. Oxidation of dyes (for example hydroethidine) to fluorescent products (e.g. ethidium bromide) can be employed for the quantification of the produced oxygen species using flow cytometry.

In this study, we evaluate the effect of core and dopant of sodium fluoride-based NCs doped with lanthanide ions on function of professional phagocytes.

## Materials and methods

### Synthesis

All the chemicals were purchased from Sigma-Aldrich and used as received without further purification. The synthesis of hexagonal in-phase yttrium and gadolinium core (both UV excited) lanthanide-doped fluoride NCs in trioctylphosphine oxide (TOPO) is based on our previous report (Banski et al. 2013). In brief, to a three-necked flask containing TOPO (90%), which serves both as a solvent and a surface ligand, we add appropriate precursors, namely sodium trifluoroacetate Na(CF_3_COO) and desired mixtures of lanthanide (III) trifluoroacetate hydrate Ln(CF_3_COO)_3_·xH_2_O (Ln = Y, Gd, Eu). The obtained mixture is then degassed by heating to 100 °C under vacuum with magnetic stirring and kept this way for about an hour, in which time TOPO turns into liquid phase. Then, the atmosphere is switched to nitrogen and the solution is heated even further up to 340 °C and left for another hour. After that, the NCs are precipitated with ethanol, centrifuged, washed several times with ethanol to remove excess TOPO, and finally dispersed in cyclohexane.

For the gadolinium core NIR samples, we used modified single-step co-thermolysis method described elsewhere (Noculak et al. 2014). In short, Gd_2_O_3_ and Tm_2_O_3_ were dissolved in trifluoroacetic acid and nitric acid, respectively. The as-obtained precursor mixture was added to oleic acid (OA) and octadecane (ODE), degassed at 120 °C while being magnetically stirred. The temperature was increased to 300 °C for 1 h and then cooled to 70 °C. The excess acetone was added to precipitate the NPs, and finally, the resulting material was collected by centrifugation.

### Functionalization

For the purpose of transferring NPs from non-polar solvents to polar, namely water, the modified ligand exchange method was employed (patent pending number P408279). In brief, around 15 mg of meso-2,3-dimercaptosuccinic acid (DMSA) was solved in 2 ml of dimethyl sulfoxide (DMSO) and mixed with 0.5 ml of NP toluene solution. The mixture was stirred for 6 h under neutral conditions (no oxygen) and centrifuged. Supernatant was discarded; NPs were solved in distilled water and sonicated for 10 min.

### Optical investigation

Photoluminescence (PL) spectra for the UV NCs have been obtained by excitation from a 450-W Xenon lamp coupled to monochromator (Triax 180, Horiba JY) and detected with HR4000 spectrometer (Ocean Optics, Dunedin, FL, USA). For the up-conversion measurement, a 980-nm 1-W laser (Shanghai Dream Lasers Technology SDL-980-LM-1000 T) was used. Signal was detected with HR4000 spectrometer.

### Structural analysis

Transmission electron microscopy pictures were taken by FEI Tecnai G2 20 X-TWIN microscope (FEI Corp., USA).

### Subjects

Eight volunteers participating in the study signed an informed consent approved by the Ethical Committee of the Slovak Medical University in Bratislava. Blood was collected by venipuncture using heparinized tubes.

### Preparation of NPs for cell treatment

NPs were vortex-shaken in the tubes for a few minutes before use and diluted with the RPMI 1640 medium containing 10% FCS to obtain a stock solution (75 μg/cm^2^). Serial dilutions of this solution in the cell culture medium were prepared to obtain the full concentration range of NP dispersions: 0.12, 0.6, 3, 15, and 75 μg/cm^2^ corresponding to 0.17, 0.85, 4.24, 21.21, and 106 μg of particles/ml, respectively.

### Phagocytic activity and respiratory burst of leukocytes

One hundred and seventy five microliters of human heparinized whole blood diluted 1:1 in complete RPMI 1640 (Sigma-Aldrich) medium containing 10% foetal calf serum (PAA), L-glutamine (Sigma-Aldrich), gentamicin (Sandoz) was dispensed into wells of a 96-well microtiter culture plate under sterile conditions. NPs were added in a volume of 25 μl. Cells were exposed to NPs for 24 h. After incubation, 30 μl of blood from each microplate well was pipetted into tube, and 10 μl of hydroethidine solution (Sigma) was added. Samples were incubated for 15 min at 37 °C. Three microliters of fluorescein-labelled *S. aureus* bacteria (1.4 × 106 per test) (Molecular Probes) were added to the “test” tubes. All tubes were incubated for another 15 min at 37 °C. Samples were put on ice and 700 μl of cold lysis solution was added. To the “control” tubes, the *S. aureus* were added after the lysis solution. Samples were tested in duplicates and analysed by flow cytometry within 30 min. Interference of NPs with the assay was tested by measuring of the same control tubes without NPs before and a few seconds after addition of NPs.

### Statistical analysis

SPSS 16.0 software was used for statistical analysis. Duplicates from each individual were averaged and used as a single value for analysis. Normality was tested by Shapiro-Wilcoxon’s test. To test for significant differences between groups, the paired-samples *T* test for normally distributed data and the Mann-Whitney U test (or Wilcoxon test) for non-normally distributed data were used. Differences between three groups were tested by one-way analysis of variance (ANOVA) and by Bonferroni’s test if equal variances were assumed or by Tamhane’s test if equal variances were not assumed. The Kruskall-Wallis test was used for non-normally distributed data. The data were expressed as mean values with standard error of mean (means + SEM). Differences at *P* < 0.05 were considered to be statistically significant.

### Samples’ description

## Results

### Optical investigation

**Fig. 1:**
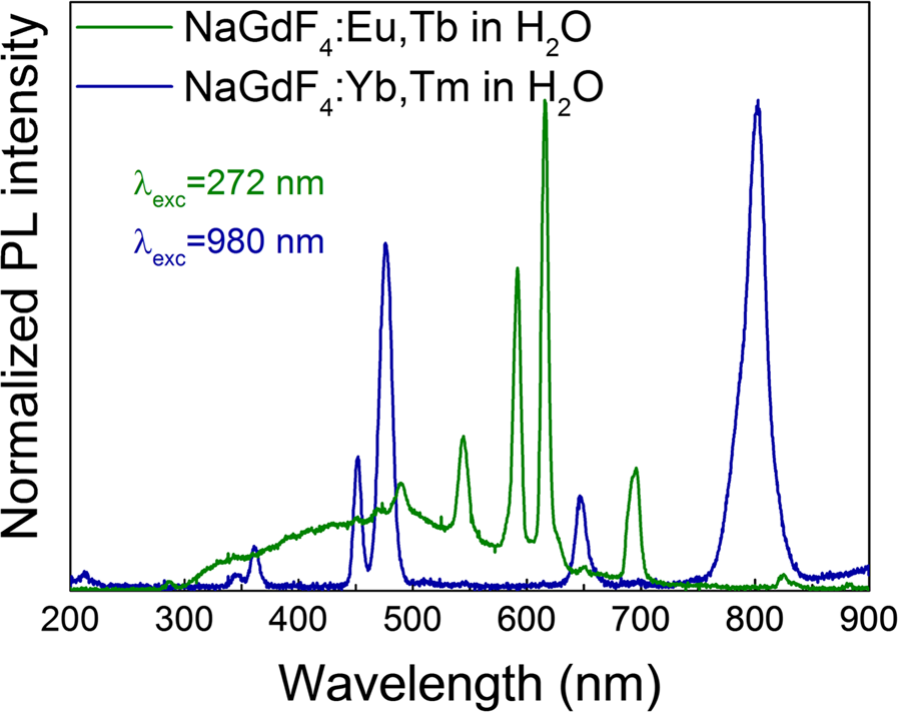
Photoluminescence spectra of hydrophilic UV (*green*) and NIR (*blue*) excited nanocrystals

In Fig. 1, the emission in VIS spectral range for both excitation types of NPs is presented. This result is obtained for samples solved in water, proving that after the surface functionalization process, the NPs preserved their photoluminescence properties. Different excitation wavelengths were used for Gd-core and Y-core to measure the PL. In particular, we used 272 or 395 nm for UV and 980 nm for NIR excited NPs.

### Structural properties

**Fig. 2:**
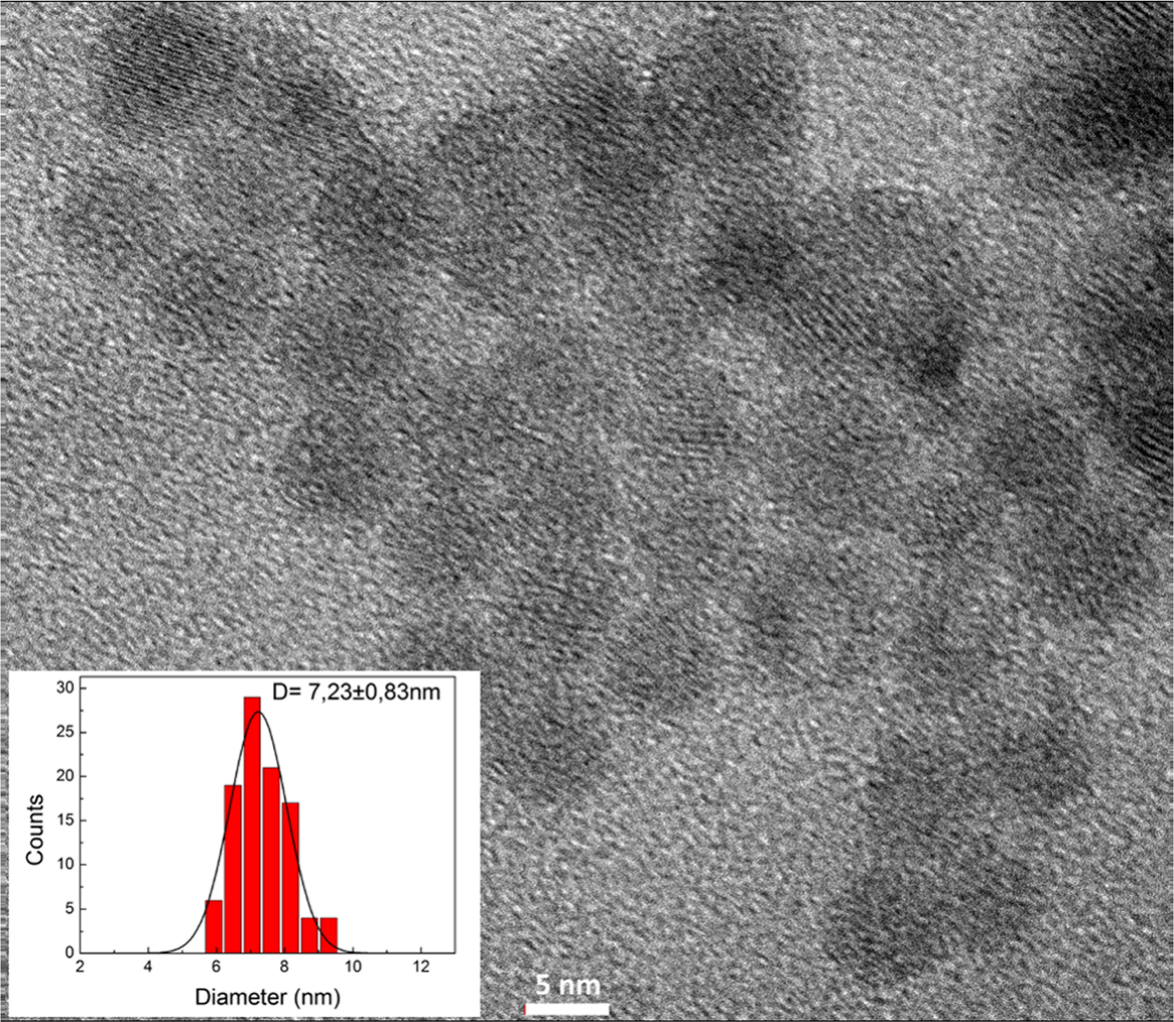
Exemplary TEM picture of samples after functionalization. Inset: nanoparticles’ size distribution

TEM picture of NPs after functionalization is presented in Fig. 2. Their morphology is intact and individual size not changed and remains under 10 nm (inset). However, after being stored for some time, they required sonication before further use, because they tend to form aggregates.

### The effect of nanoparticles on phagocytic activity and respiratory burst of leukocytes

**Fig. 3:**
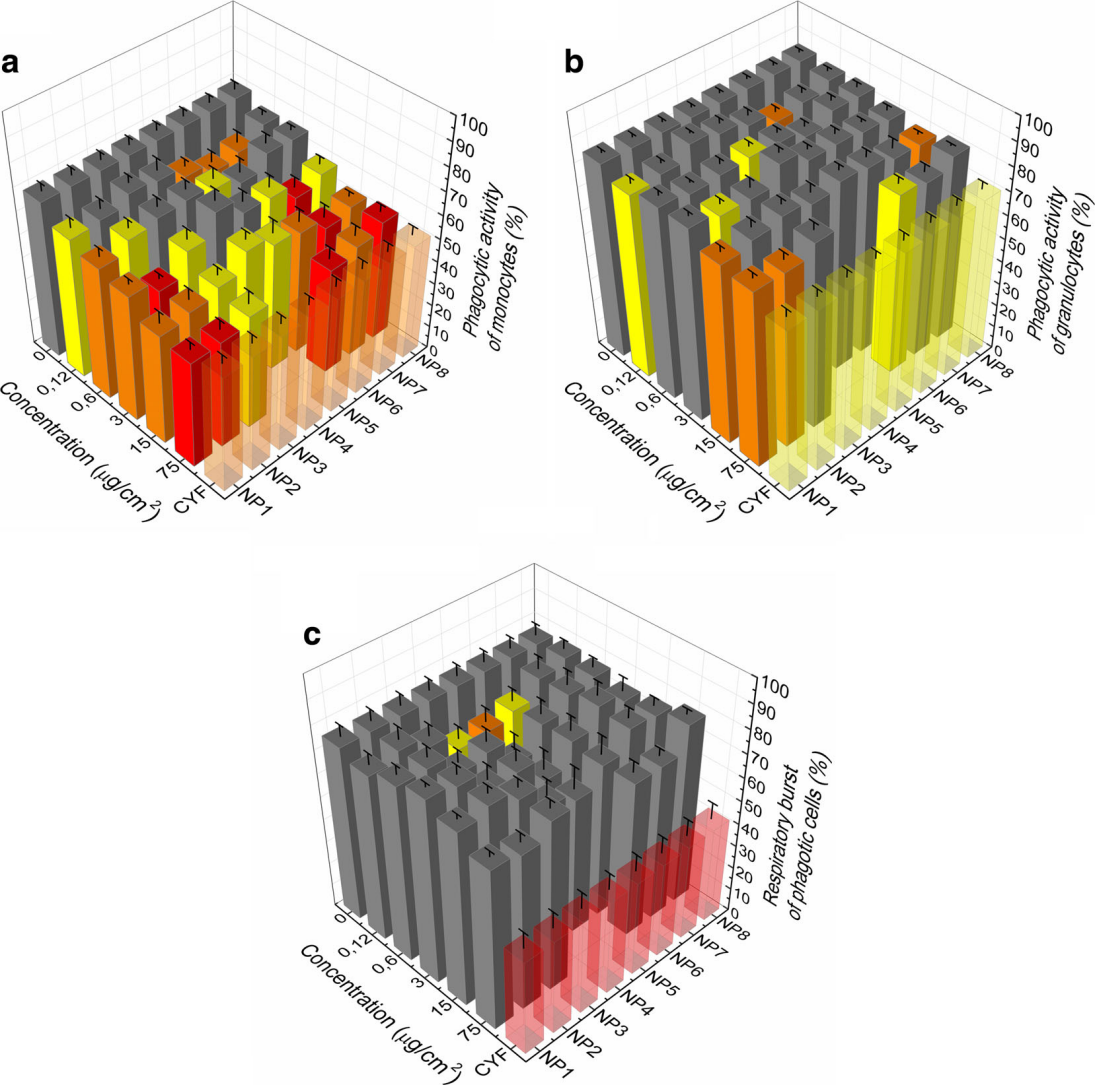
The effect of sodium fluoride-based nanocrystals doped with lanthanide ions on phagocytic activity of monocytes (**a**) and granulocytes (**b**) evaluated using ingestion of fluorescein-labelled *Staphylococcus aureus* and the respiratory burst (**c**) monitored using hydroxyethidine by flow cytometry. Results are expressed as percentage of phagocytic activity and respiratory burst (mean + SEM). *Bars* indicate mean group activity in peripheral blood cultures in vitro treated with different concentrations of NPs: 0 μg/cm^2^ (control), 0.12 μg/cm^2^, 0.6 μg/cm^2^, 3 μg/cm^2^, 15 μg/cm^2^, 75 μg/cm^2^, CYF—suppressive control exposed to cyclophosphamide 40 mg/ml. The assay was performed after 24 h in vitro exposure of the peripheral blood cells (*n* = 8 human volunteers). Statistical significance: *p < 0.05 (*red*), **p < 0.01 (*orange*), ***p < 0.001 (*yellow*)

Experiments were performed with eight different hydrophilic sodium fluoride-based NCs. Two of the NPs were prepared with Y-core and six with Gd-core. Y-core-based NCs were doped with Eu ions, which enabled them to be excited with UV light wavelengths. Gd-core NPs were doped with combination of Eu and Tb lanthanide ions in two different concentrations as the UV excitation type. On the other hand, doping them with ytterbium and thulium resulted in the NIR excitation type (Table 1). Five concentrations of NCs, one vehicle and one suppressive control (cyclophosphamide) were tested in eight samples of human blood, and tests were performed in duplicate tubes. Means ± standard error mean (SEM) were calculated. No interferences with the assay were found during the testing of NPs in experiments. The phagocytic function of monocytes (Fig. 3a) and granulocytes (Fig. 3b) was evaluated using ingestion of fluorescein-labelled *S. aureus*, and the respiratory burst (Fig. 3c) was monitored using hydroxyethidine.

**Table 1:** List of nanoparticles used in this work

NP name	Core type	Dopant	Exact crystal structure	Excitation type	Functionalization time
NP1	Y-core	Eu(2%)	NaYF_4_	UV	21 h
NP2			NaYF_4_		18 h
NP3	Gd-core	Tm(1%) Yb(10%)	NaGdF_4_	NIR	18 h
NP4			NaGdF_4_		21 h
NP5		Eu(5%) Tb(2%)	NaGdF_4_	UV	18 h
NP6		Eu(5%) Tb(10%)	NaGdF_4_		18 h
NP7			NaGdF_4_		16 h
NP8			NaGdF_4_		8 h

### The effect of the core

Although half of both core types (Y, Gd) affected the phagocytic activity of monocytes exposed even to low dose of NPs, one sample of Gd-core-based NIR-excited NPs (4) did not show any toxicity to the function of cells treated up to 3 μg/cm^2^ (4.24 μg/ml). Generally, phagocytic activity of granulocytes was affected less markedly than were functions of monocytes. Decreased phagocytic activity of granulocytes was seen mostly in cells exposed to higher doses of NPs. Exception was one representative of the both Y- and Gd-core groups (1,6) which suppressed phagocytic function already in cells treated with low dose (0.12 μg/cm^2^, e.g. 0.17 μg/ml) of NCs. However, no dose-dependent effect was recorded; since suppression was not found in cells treated with higher doses (0.6 or 3 μg/cm^2^) of NCs. When comparing the effect of core, there is, however, one noteworthy difference, namely that the Y-core NPs seem to have a stronger effect on phagocytic activity of granulocytes than Gd-core NPs. Respiratory burst of cells was suppressed by both core types similarly, surprisingly mostly in cells treated by low doses of NPs.

### The effect of the dopant

#### The effect of UV-excited Y-core NPs

For these NCs, clear dose dependence is observable for the inhibition of phagocytic activity of monocytes. Moreover, for the highest dose, a toxic influence can be noted for the granulocytes as well. Respiratory burst of granulocytes was less affected and no alteration was found.

#### The effect of NIR-excited Gd-core NPs

The impact of NIR-excited Gd-core NPs on phagocytic activity of monocytes is less pronounced than the effect of their UV-excited counterparts. Similarly, the phagocytic function of granulocytes was less affected; therefore (for now), one can conclude that the dopant in the NCs may be of some significance when it comes to toxicity of the whole NP.

#### The effect of UV-excited Gd-core NPs

For the NaGdF4/Eu (5%), Tb (2%) sample (5), there is no clear dose-dependent effect on any of the examined parameters within the conducted test. However, for this sample, there are some statistically significant decreases in phagocytic activity of monocytes and respiratory burst of phagocytes observable for the low selected doses: 0.12 and 0.6 μg/cm^2^ (0.17 and 0.85 μg/ml). Since this phenomenon occurs in a number of other samples, it will be discussed in the next paragraph. From the remaining three NaGdF4/Eu (5%), Tb (10%) samples (6, 7, 8), a clear dose-dependent suppression in the phagocytic activity of monocytes is observable, mainly for sample 8. Phagocytic activity of granulocytes was less affected but in few cases significantly inhibited without dose-dependent response. Respiratory burst of cells displayed no toxic effects, but in one case (6), it appeared in cells exposed to the low dose of NCs.

#### Comparison of all UV-excited NPs

It is worth mentioning that all of the UV-excited samples suppressed the phagocytic activity of monocytes. In the case of phagocytic activity of granulocytes, Y-core NCs seem to have greater impact on decrease in phagocytic activity of cells. All UV samples, except nos. 5 and 6, did not differ with respect to the respiratory burst of phagocytic cells.

### Combined effect

Summing up all the results for different samples of both types of NCs, the sensitivity of phagocytosis to exposure to NCs can be expressed: monocytes > granulocytes > respiratory burst. Toxicity of NCs for monocytes decreases in the following order: Gd-core UV > Y-core UV > Gd core NIR (NP no. 6 = 7 > 1 > 2 > 5 > 3 = 8 > 4). For granulocytes, the situation is a little different: Gd-core UV (6) = Y-core UV (1) > Gd-core NIR (4) > Y-core UV (2) > Gd-core UV (8). The toxicity of NCs on respiratory burst can be summarized as follows: Gd-core UV > Gd-core NIR > Y-core UV (5 > 4 > 6). The lowest toxicity was observed in sample nos. 3 and 4 (Gd-core, Yb, Tm dopants, NIR NCs). Clear dose-dependent effect of NCs on the phagocytic activity of leukocytes and the respiratory burst of cells was observed for a limited number of samples.

## Discussion

Our results indicate that, in general, hydrophilic sodium fluoride-based NCs doped with lanthanide ions have very low toxicity to granulocytes from the phagocytosis point of view with exception of few cases. This is in agreement with our previous work (Sojka et al. 2014), in which we suggested that the main reason for toxicity was the solvent—cyclohexane—and once NPs would be hydrophilic, this should no longer be an issue.

The effects of sodium fluoride-based NCs were more pronounced on phagocytosis in monocytes than in granulocytes, and phagocytosis was more affected than respiratory burst. The different responses in granulocytes and monocytes might be explained by different cell sensitivity to the toxicity of the NCs and more work need to be performed to understand that mechanism.

Statistically significant suppression in exposed cells was found, for half of the cases without clear dose dependence. We have noticed that in some cases, there is a statistically significant decrease in phagocytic function for the smallest dose of NCs and it is present regardless of any of the three tested parameters. We presume it is due to the fact that in the smallest dose (concentration), the NC aggregates are smaller, because there is not enough nanomaterial to make them as big as they are in higher doses. The aggregate size difference leads to a different surface-to-volume ratio, and this results in a different amount of available active surface per the NCs dose. In other words, in smaller aggregates, more NCs compared to their overall number are at the surface of the aggregate (higher surface-to-volume ratio) than in bigger ones (lower surface-to-volume ratio). This means more NCs are able to interact with leucocytes and thus probably influence their phagocytic activity more than their alleged concentration/dose would suggest. The choice of lanthanide dopant also seems to play a role. Firstly, during comparison of the Y-core and Gd-core NCs, the latter had a smaller advantage. Secondly, NIR-excited NCs are just a little more favourable, from the application point of view, over UV excited. Therefore, we presume the least toxic NCs would be NIR excited with Gd-core. We suggest more tests should be conducted in the future on such NCs with careful examination on their size (different surface-to-volume ratio).

Published data on the safety assessment mostly indicate low toxicity of lanthanide NCs. For example, the in vitro studies with human cervical cancer cells (HeLa) and in vivo studies with worms (*Caenorhabditis elegans*) published by Zhou et al. (2011) confirmed low in vitro cytotoxicity and no obvious in vivo toxicity of NaYF_4_/Yb,Tm NCs which could serve as a NIR emission bioprobe. On the other hand, some studies displayed toxic effect of rare earth NCs on various cell functions. Results on the long-term cytotoxicity of lanthanide-doped NPs (NaGdF_4_ and NaYF_4_) in HeLa cells showed induction of intracellular ATP deprivation, resulting in a significant decrease in cell viability after exposure for 7 days (Tian et al. 2015). Chen et al. (2014) reported that europium-doped NaYF_4_/Eu^3+^ could be up taken into endothelial cells (human umbilical vein cell line) and decrease the cell viability, induce the intracellular LDH, increase the ROS level and decrease the cell mitochondrial membrane potential (MMP) in a size-dependent manner. Besides that, not only did the cells suffer apoptosis with the caspase-3 activation, but it also increased the inflammation of specific gene expressions (ICAM1 and VCAM1). Das et al. (2014) showed that toxicity of the oleate-capped hydrophobic NaYF_4_/Yb,Er NPs with three different modified surfaces on primary human aortic endothelial cells depends strongly on ligand coordination conditions, in addition to the size, structure, and surface charge. Wysokińska et al. (2016) demonstrated the role of coating in modulation of toxicity. While bare NaGdF_4_/Yb^3+^:Er^3+^ NCs were cytotoxic and induced apoptosis of both mouse embryonic fibroblasts (NIH3T3) and mouse leukemic monocyte-macrophage cells (RAW264.7), adverse effect was reduced by PEGylation and coating with silica which seems to be reasonable approaches to decrease cytotoxicity of the NCs.

To our knowledge, no published data on the effect of lanthanide NCs on phagocytic activity and respiratory burst of human leukocytes are available. However, the effect of other kinds of NPs on phagocytic function was broadly examined with different results. Immune effect in phagocytes was examined either after in vivo treatment of animals (ex vivo) or by in vitro treatment of cultivated cells, mostly cancer cell lines, by testing of phagocytosis, respiratory burst (reactive oxygen production), cytokine secretion (proinflammatory response), chemotaxis, NO generation, etc.

Published findings indicate ability of NPs to modulate phagocytic functions in different ways. Example of NPs “non-interfering” with phagocyte function are iron oxide particles (Ferumoxtran-10) approved for medical use, which did not influence the different aspects of phagocyte function. The secretion of proinflammatory cytokines, oxidative burst, phagocytosis and chemotaxis is not affected by the exposure to the particles in vitro (Müller et al. 2007). The effect may change with different coating as demonstrated by Strehl et al. (2015) who found no effect of amino-polyvinyl alcohol coated superparamagnetic iron oxide NPs (a-PVA-SPIONs) on the viability of human immune cells, but cytokine secretion was affected. Percentage of viable macrophages was increased after exposure to a-PVA-SPIONs. This effect was even stronger when a-PVA-SPIONs were added very early in the differentiation process.

Significant stimulation of phagocytic function was observed in several studies. Małaczewska (2015) studied the effect of oral administration of commercial gold nanocolloid on peripheral blood leukocytes in mice and found elevated activity of granulocytes and monocytes, in terms of both phagocytic and respiratory burst activity. Changes were transient and noticed only after a short time of administration. After in vitro exposure of macrophages to gold NPs, proinflammatory response with induction of IL-6 and TNF-α release with no significant impact on macrophage viability was found (Brown et al. 2014). Proinflammatory cytokine secretion increased respiratory burst, or neutrophilic granulocyte activation was also observed after in vitro exposure to polystyrene, silver, SiO_2_ or TiO_2_ NPs (Prietl et al. 2014, Haase et al. 2014, Shin et al. 2007, Carlson et al. 2008). ).

Significant suppression of phagocytic function was observed in several studies. Aude-Garcia et al. (2016) showed that zinc oxide NPs induced specifically a strong decrease in phagocytosis and the mitochondrial function and an increase in the methylglyoxal-associated DNA damage in macrophage cell line (J774). Wang et al. (2014) demonstrated that possible mechanisms of ZnO toxicity in RAW 246.7 cells induced elevation of intracellular Zn^2+^ concentration, leading to the over generation of intracellular ROS, leakage of plasma membrane, dysfunction of mitochondria and cell death. Similarly, silver wires induced ROS generation, lysosomal rupture, cathepsin B, caspase-1 and IL-1β production in human acute monocytic leukaemia (THP-1) cells (Jung et al. 2014).

## Conclusions

In this work, lanthanide-doped sodium fluoride NCs were synthesized and surface modified in order to be solved in water. They preserved their structural and optical properties and could be potentially used as biomarkers for imaging. To evaluate this statement, their influence on phagocytic activity and respiratory burst of human peripheral blood leukocytes was estimated. It concluded that neither type of examined NPs induces toxic effects within the parameters of the conducted test, although some samples produced suppressive effects in selected doses tested mostly without clear dose-dependency. Both core types of NCs (Y-core and Gd-core) impaired the phagocytic activity of monocytes the strongest, having minimal or none whatsoever influence on granulocytes and respiratory burst of phagocytic cells. From the intended application point of view, as well as from the obtained results, the best sample is NIR excited with gadolinium core.
